# Effectiveness and safety of medication abortion with vs without screening ultrasonography or pelvic examination

**DOI:** 10.1016/j.ajog.2025.06.013

**Published:** 2025-06-19

**Authors:** Leah R. Koenig, Elizabeth G. Raymond, Ushma D. Upadhyay, Laura J. Frye, Bliss Kaneshiro, Christy M. Boraas, Catherine E. Oldenburg, David V. Glidden

**Affiliations:** Department of Epidemiology and Biostatistics, University of California, San Francisco, CA; Advancing New Standards in Reproductive Health, Department of Obstetrics, Gynecology & Reproductive Sciences, University of California, Oakland, CA; Center for Gender and Health Justice, Global Health Institute, University of California, Oakland, CA; Department of Obstetrics and Gynecology, University of Washington, Seattle, WA; Advancing New Standards in Reproductive Health, Department of Obstetrics, Gynecology & Reproductive Sciences, University of California, Oakland, CA; Center for Gender and Health Justice, Global Health Institute, University of California, Oakland, CA; Gynuity Health Projects, New York, NY; Department of Obstetrics and Gynecology, University of Hawaii, Honolulu, HI; Planned Parenthood North Central States, Minneapolis, MN; Department of Epidemiology and Biostatistics, University of California, San Francisco, CA; Francis I Proctor Foundation, University of California, San Francisco, San Francisco, CA; Department of Ophthalmology, University of California, San Francisco, CA; Department of Epidemiology and Biostatistics, University of California, San Francisco, CA

**Keywords:** medication abortion, no-test medication abortion, screening pelvic exam, screening ultrasound

## Abstract

**BACKGROUND:**

Before the COVID-19 pandemic, clinicians in the United States routinely required in-person screening tests such as ultrasonography or pelvic examination for medication abortion care. To minimize physical contact during the pandemic, clinicians began providing “no-test” medication abortion care, which can improve access by facilitating care from a broader range of clinicians, lowering costs, and reducing wait times. Despite evidence that medication abortion without in-person screening tests is safe and effective, screening ultrasound and pelvic examination remain common when medication abortion care is provided in person.

**OBJECTIVE:**

In this cohort study, we compared the effectiveness and safety of medication abortion provided with and without in-person screening tests, specifically ultrasonography or pelvic examination.

**STUDY DESIGN:**

We conducted a retrospective cohort study of medication abortions provided as a part of regular clinical practice by 3 US clinics from February 2020–January 2021. Clinics abstracted medical record data for all patients with gestations <77 days who had neither screening ultrasonography nor pelvic examination (“no-test” group) and a random sample who had either test (“screening test” group). Participating clinics provided medication abortion care both with and without screening tests during the study period, and patients were screened either in person or via telehealth in both study groups. We imputed missing data and used inverse-probability-of-treatment-weighted logistic regression to compare abortion effectiveness (complete abortion without additional treatment), safety (abortions not involving major adverse events), emergency department visits, and abortions inadvertently provided beyond 77 days (a commonly used pregnancy duration limit for medication abortion) between study groups.

**RESULTS:**

We included 649 abortions with and 1727 abortions without screening tests. Patients who obtained medication abortion without screening ultrasonography or pelvic examination had lower average pregnancy durations (48 vs 50 days, *P*<.001), were more likely to be White (43% vs 32%, *P*<.001), were less likely to reside in urban areas (78% vs 83%, *P*=.004), were more likely to receive abortion medications by mail (35% vs 1%, *P*<.001), and were more likely to pay out-of-pocket for abortion costs (62% vs 55%, *P*=.002). Abortion outcomes were documented for 72% of the screening test group and 58% of the no-test group (*P*<.001). After imputing missing outcome data, we found similar effectiveness (95.3% vs 93.4%; risk difference, −1.9% [95% confidence interval, −6.9%, 3.1%]) and safety (99.8% vs 98.7%; risk difference, −1.1% [95% confidence interval, −2.9%, 0.7%]) between the screening test and no-test groups. Complete case analyses also found similar effectiveness (95.8% vs 94.1%; risk difference, −1.7% [−4.4%, 1.1%]) and safety (100.0% vs 99.2%; risk difference, −0.81% [−1.74%, 0.11%]) between study groups. Across both groups, 0.59% of abortions were found at follow-up to have been inadvertently provided at >77 days of gestation and 4.0% involved an emergency department visit; these proportions did not differ by study group. Emergency department visits involving treatment were more common in the no-test group (2.1% vs 0.39%; risk difference, 1.7% [95% confidence interval, 0.1%, 3.3%]).

**CONCLUSION:**

This study indicates that medication abortion care provided without screening ultrasonography or pelvic examination is comparably safe and effective to care with these screening tests. Although loss to follow-up was high, results are consistent with previous studies. Medication abortion should be routinely offered without screening ultrasonography or pelvic examination to eligible patients, including those screened in person.

## Introduction

Until the COVID-19 pandemic, standard clinical practice for medication abortion in the United States included pretreatment screening with ultrasonography or pelvic examination. During the pandemic, clinicians began to provide “no-test” medication abortion to reduce physical contact. In this new care model, screening relied on patient history rather than in-person screening tests.^1^

“No-test” telehealth medication abortion first became widely available in the United States in 2021 when the US Food and Drug Administration lifted an in-person dispensing requirement on mifepristone, the first drug in the medication abortion regimen. Since then, no-test telehealth medication abortion care has expanded, especially since the 2022 US Supreme Court *Dobbs v. Jackson Women’s Health Organization* decision ended a constitutional protection for abortion.^2^ However, most medication abortions are still provided with in-person screening tests, due to policy and clinical barriers.^3^ Among the states where abortion remains legal, 7 (AZ, FL, GA, IA, NC, SC, and WI) require ultrasonography before all abortions.^4^ Additionally, screening protocols for in-person medication abortion have been slow to charge.^3,5^ Omitting unnecessary in-person screening tests can improve access to medication abortion care by allowing providers without ultrasound technology, such as community clinics and other primary care providers, to offer care, ultimately reducing costs and wait times.

Mounting evidence suggests that these tests may not be medically necessary for most patients and that omitting them can improve access to care without compromising safety or effectiveness. However, most studies did not include comparison groups that received screening tests concurrently, in the same population.^6–10^ Only 3 small US studies have directly compared medication abortions provided with and without these tests. They determined that both models were effective and safe.^11–13^ One found that patients in 2020 who did not have screening tests were more likely than those who obtained screening tests to receive procedural intervention and to have unplanned clinical encounters, although this difference did not persist into 2021.^14^ More research is needed to assess the comparability of these models of care to inform change in clinical practice.

We previously published a retrospective case-series study examining outcomes of “no-test” medication abortion, defined as medication abortions provided without ultrasonography or pelvic examination, in 2020 to 2021.^6^ While we found high effectiveness and safety, this study lacked a comparison group. To address this limitation, we subsequently collected data from 3 of the participating clinics for medication abortion patients who had screening tests during the same period. We aimed to compare the effectiveness and safety of the screening test and no-test medication abortion care models.

## Materials and methods

### Data

For this study, we drew data from a prior study of 3779 “no-test” medication abortions, defined as those provided without screening ultrasonography or pelvic examination, from 14 clinics.^6^ The present study includes the clinics in the original study that contributed more than 50 “no-test” cases to the original study, provided medication abortions with and without these tests during the original study period, and were able to participate in continued research. We originally planned to abstract 1000 abortions with screening tests across 7 clinics; however, only 3 clinics were ultimately able to participate. Medical eligibility for no-test abortion, based on a published protocol,^1^ included a known first date of last menstrual period and absence of risk factors for ectopic pregnancy (eg, sterilization, prior ectopic pregnancy, or intrauterine device in place, pelvic inflammatory disease).

For the analysis, we included all abortions in the original study (all of which were provided without ultrasonography or pelvic examination) from these 3 clinics in which the estimated pregnancy duration was <77 days (a common pregnancy duration limit among medication abortion providers^15^) at treatment; the patient had not had a prior qualifying abortion during the study period; the abortion was not provided within the TelA-bortion study, a clinical trial on telehealth abortion (as we anticipated outcomes may have differed within the context of a clinical trial and outcomes of these patients were published previously)^10,12^; and the patient took mifepristone, misoprostol, or both.^6^ These cases constituted the “no-test” group. For comparison, we developed a “screening test” group comprised of patients who met the same criteria except that the patient had screening ultrasonography or pelvic examination. We created a sampling frame that was stratified by clinic and included all eligible patients between February 2020 and January 2021. We selected screening test group cases using a random sequence until the target enrollment number, proportional to the distribution across clinics in the original study, was reached for each site.

Patients in this study obtained abortions during the height of the COVID-19 pandemic when many clinics were offering no-test medication abortion to increase social distancing and reduce COVID-19 transmission risks. Each participating clinic screened some patients in person and some patients via telehealth. However, the screening modality for each patient was not abstracted. We did obtain case-specific data on whether the patient received abortion medications in person or by mail, and in both groups, most patients picked up their medications in person.

Trained study team members abstracted electronic medical record data into a standardized form in Research Electronic Data Capture.^16^ This study was approved by the Allendale and University of Hawaii institutional review boards, which waived the need for patient informed consent given the retrospective medical record review study design. The study was also deemed exempt by the University of California, San Francisco Institutional Review Board.

### Measures

Outcomes were ascertained from all follow-up interactions (either remote interactions or in-person visits) documented in patients’ electronic medical records.

#### Effectiveness

We compared the proportion of medication abortions with known outcomes that were complete with 200 mg mifepristone and 1600 mcg misoprostol without other intervention between study groups. We used a threshold of 1600 mcg of misoprostol to reflect the maximum amount of misoprostol dispensed to patients at the time of treatment. We classified abortions as complete by test if the patient had a negative urine pregnancy test result, ultrasonography or pelvic examination showing no continuing pregnancy, an expected decline in serum beta human chorionic gonadotropin level, a single post-treatment serum beta human chorionic gonadotropin value less than 500 mIU/mL ≥8 days after mifepristone dispensing, or clinician examination of fetus or fetal parts. Patients without a negative test were considered to have a complete abortion determined by history if their provider assessed symptoms as consistent with a complete abortion or reported no concern that additional evaluation or management was necessary.

We considered patients to not have had a complete abortion if they experienced an ultrasonography-confirmed continuing pregnancy at any point after initial treatment and/or intervention to complete the abortion. Interventions included (1) a procedure such as an aspiration; (2) a prescription of >200 mg mifepristone, >1600 mcg misoprostol, or an uterotonic medication to complete the abortion; or (3) treatment for ectopic pregnancy.

#### Safety

We defined major adverse events as blood transfusion, major surgery, or hospital admission. We examined safety among patients who either had known outcomes and/or known major adverse events. Among this sample, we estimated the proportion of patients who did not experience a major adverse event.

#### Other outcomes

We also examined the proportion of patients who were found at follow-up to have been treated at >77 days gestation, had emergency department (ED) visits, and had ED visits with treatment.

### Analyses

We compared sociodemographic characteristics, obstetric history, abortion characteristics, and pregnancy duration by last menstrual period date, between the study groups using *t*-tests and Fisher’s exact tests.

We used multiple imputation by chained equations with 200 imputations to impute missing outcome and covariate data (for race or ethnicity and previous medication abortion). We included patients’ pretreatment characteristics with no missing data (patient age, urbanicity, clinic, pregnancy duration, and whether mifepristone was dispensed in person or by mail) as independent variables in the imputation model.

We first calculated proportions of each abortion outcome across the sample using bivariate logistic regression. We then used logistic regression with inverse-probability-of-treatment weighting to estimate risk differences (RDs) comparing outcomes in each study group and corresponding outcome proportions within each study group.^17^ We calculated weights using patient age, race or ethnicity, pregnancy duration, providing clinic, prior medication abortions, and mifepristone dispensing method. We calculated robust standard errors, using pseudo-observations to stabilize results in scenarios with rare outcomes.^18^ Then, we conducted unimputed (complete case) versions of the primary analyses. All analyses used StataMP version 18.0 (College Station, TX).

### Sensitivity analyses

First, we reconstructed our imputed analyses only among patients whose abortion medications were dispensed in person, to account for imbalance of mifepristone dispensing methods across the study groups and potential variation in outcomes by dispensing method. Second, we replicated the imputed analyses after excluding the clinic with the lowest follow-up (45%) to assess whether the primary results held in a sample with higher follow-up. Finally, we used 2 additional missing data approaches^19,20^—multiple imputation with a monotone-missing data pattern and inverse-probability weighting—to assess the performance of our primary imputation approach.

## Results

**FIGURE 1:**
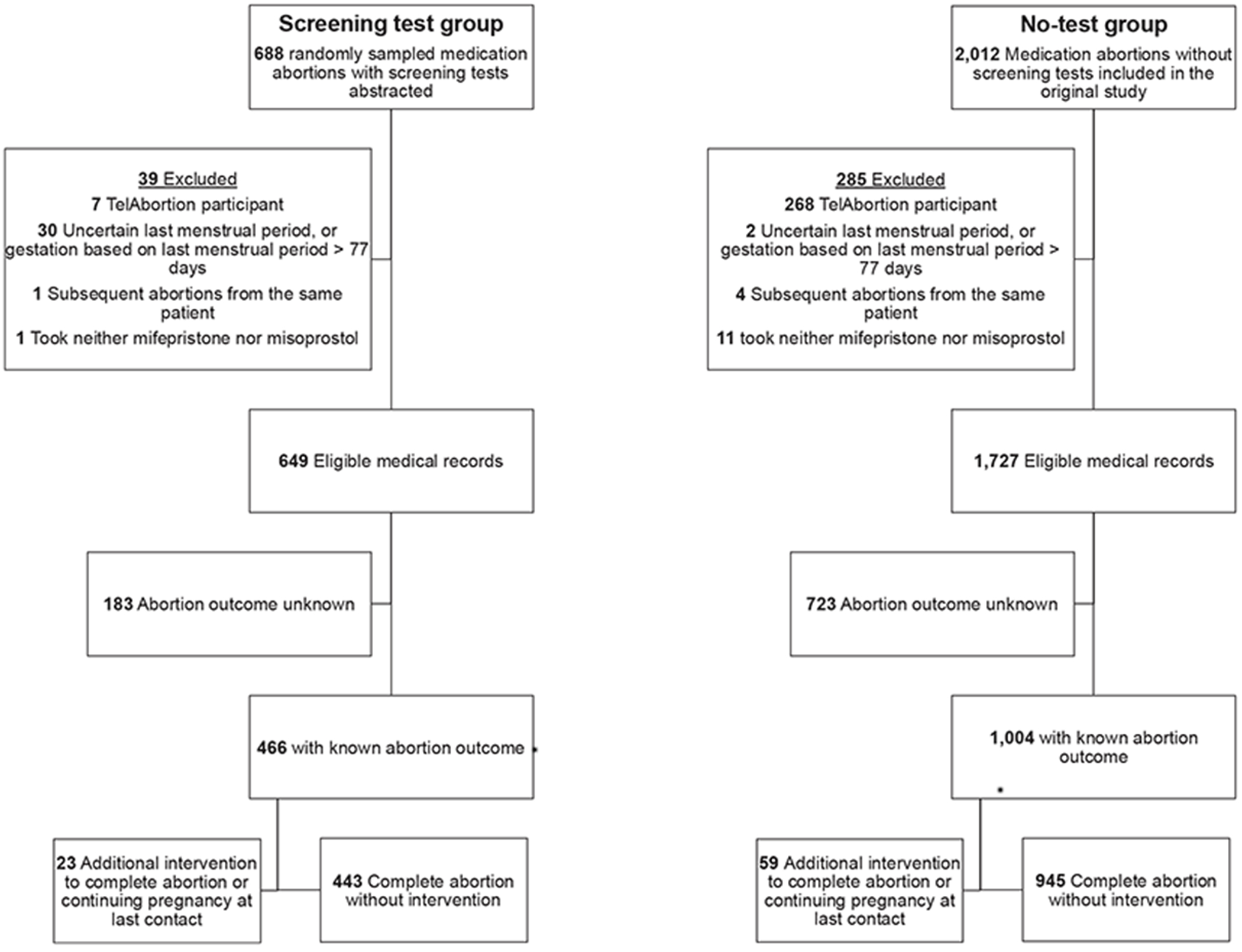
Study sample and inclusion criteria flowchart

Our sample initially included 2681 records. After applying our exclusion criteria, our analytic sample included 2376 patients: 649 (27%) who obtained screening tests and 1727 (73%) who did not obtain screening tests (Figure 1). Among those, 1470 had known abortion outcomes: 466 (72%) in the screening test group and 1004 (58%) in the no-test group (*P*<.001). In Supplemetal Figure 1, we documented that abortion outcomes were more likely to be known in the screening test group within most strata of participant and abortion characteristics.

Patient age was similar between the study groups (Table 1). Mean pregnancy duration determined by last menstrual period was slightly higher in the no-test group (50 days vs 48 days, *P*<.001). In the screening test group, nearly all (99%) obtained the mifepristone in person, while in the no-test group, 65% obtained the mifepristone in person and 35% obtained it by mail (*P*<.001). A higher proportion of patients in the no-test group were White (43% vs 32%, *P*<.001), resided in rural areas (14% vs 10%, *P*=.004), and paid out-of-pocket for abortion costs (62% vs 55%, *P*=.002).

### Effectiveness

**FIGURE 2:**
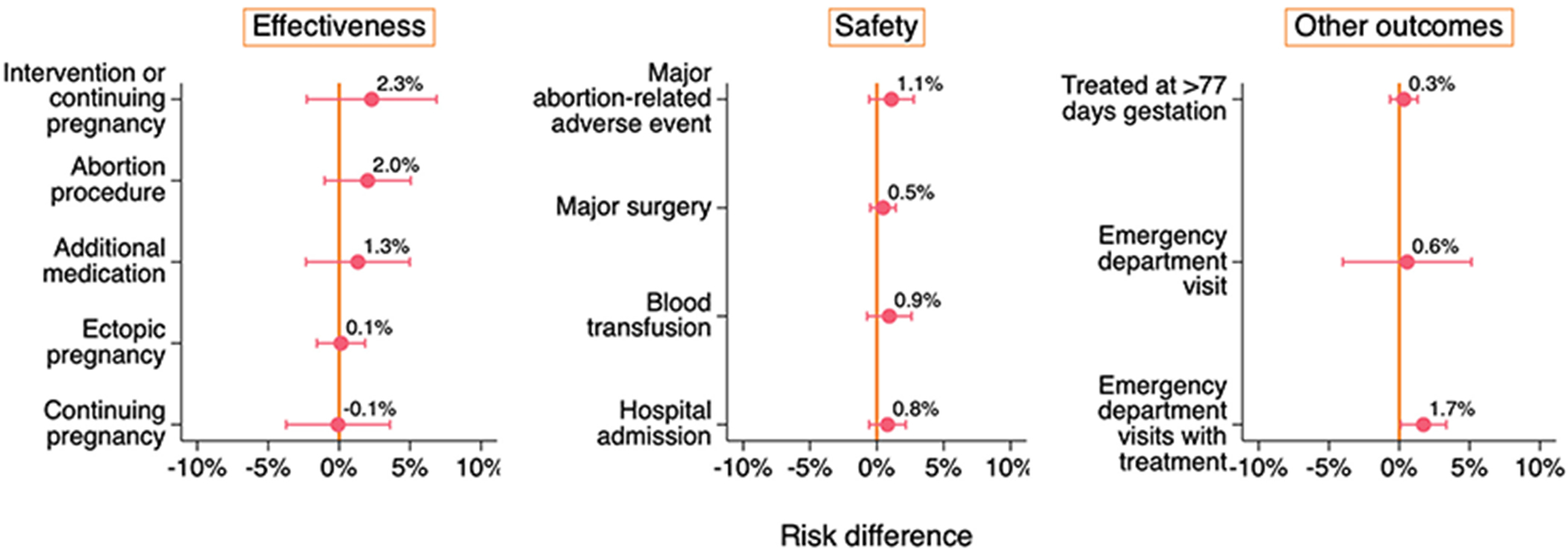
Risk differences in abortion outcomes between patients who obtained medication abortion care with and without screening tests Estimates draw from imputed logistic regression analyses with inverse-probability of treatment weights. Positive risk difference values indicate higher risk in the no-test vs. screening test group. Risks were similar by study group for most outcomes except for emergency department visits with treatment, which were more common in the no-test group.

In our imputed analyses, 93.4% (95% confidence interval [CI], 92.0%, 94.9%) of patients overall had a complete abortion without additional intervention (Figure 2, Table 2). Among those with complete abortions, we estimated that abortion outcomes were determined by history alone for 34% of those in the screening test group and 27% of those in the no-test group in our imputed analysis. Among the 6.6% of patients whose abortions were not complete without additional intervention, 4.0% had a procedure or aspiration, 2.5% obtained additional medications, 0.48% were treated for ectopic pregnancy, and 2.4% had a continuing pregnancy after initial treatment. Effectiveness (95.3% in the screening test group and 93.4% in the no-test group; RD, −1.9%, [95% CI, −6.9%, 3.1%]) and all other effectiveness outcomes were similar between the study groups (Table 2).

### Safety

In our primary, imputed analyses, we found that 99.0% (95% CI, 98.2%, 99.7%) of patients did not experience a major adverse event (Figure 2, Table 2). Overall, the proportion of patients who experienced a major adverse event was 1.0% (95% CI, 0.3%, 1.7%); 0.19% in the screening test group and 1.3% in the no-test group (RD, 1.1% [95% CI, 0.6%, 2.8%]). All safety outcomes, including blood transfusion, major surgery, and hospital admission, were similar between the study groups.

### Other outcomes

In the primary imputed analysis, 4.0% (95% CI, 2.8%, 5.1%) of patients had an ED visit following their treatment, 3.9% in the screening test group, and 4.5% in the no-test group (RD, 0.56% [95% CI, 4.01%, 5.13%]). We estimated that 1.7% of the sample had ED visits that involved treatment (40% of all ED visits involved treatment, 16% of ED visits in the screening test group, and 45% of ED visits in the no-test group). ED visits involving treatment were less common in the screening test group (0.39%) than in the no-test group (2.1%; RD, 1.7% [95% CI, 0.1%, 3.3%]). Among the 19 ED visits involving treatment, patients were most commonly treated with aspiration procedures (n=7), blood transfusion (n=6), additional medication (n=3), and pain medication (n=3, treatments were not mutually exclusive). Less common treatments included surgery, treatment for ectopic pregnancy, antibiotics, and nausea medications. Across the sample, one patient, who was in the no-test group, was found by follow-up ultrasonography to have been treated at 89 days. This patient had a continuing pregnancy after initial treatment treated with an abortion procedure at 101 days of gestation.

### Complete case analyses

In our complete case analyses (Supplemetal Table 1), 94.4% of patients overall had a complete abortion without additional intervention and 99.5% did not experience a major adverse event. All outcomes were similar between the 2 study groups; however, patients in the screening test group were less likely to be prescribed additional medications compared to the no-test group (0.59% vs 2.1%; RD, 1.5% [95% CI, 0.1%, 2.9%]). ED visits involving treatment were less common in the screening test group compared to the no-test group (0% vs 1.7%; RD, 1.7% [95% CI, 0.6%, 2.7%]).

### Sensitivity analyses

In the first sensitivity analysis that restricted our imputed analysis only to patients who obtained their abortion medications in person, we estimated that 95.6% of patients overall had a complete abortion without additional intervention, 99.3% did not experience a major adverse event, and no outcomes differed by study group (Supplemetal Table 2).

In the second sensitivity analysis, we excluded the one clinic with very low follow-up, after which follow-up was 76% in each group (*P*=.942). In this analysis, we estimated 96.2% of patients overall had a complete abortion without additional intervention, 99.3% did not experience a major adverse event, and no outcomes differed by study group (Supplemetal Table 3).

Accounting for missing abortion outcome data using 2 additional approaches yielded results consistent with the primary imputed analysis (Supplemetal Table 4 and 5).

## Comment

### Principal findings

In this study, we found that medication abortion provided without screening ultrasonography or pelvic examination was comparably effective and safe to medication abortion with those tests. We estimated that 93.4% of all abortions were complete without additional intervention and 99.0% were not followed by a major adverse event. Even after imputing missing abortion outcome data, these outcomes did not differ by study group.

### Results in the context of what is known

The effectiveness and safety estimates in this study are comparable to evidence on medication abortion with^21–24^ and without^3,7–9^ in-person screening tests in the published literature. Like prior direct comparisons of these models, our study found few differences in effectiveness and safety.^11,13,14^ This analysis is the largest study to our knowledge to make this comparison and applied rigorous statistical methods to minimize bias.

We observed differences in the characteristics of patients who obtained medication abortion care with and without screening tests. No-test patients were more likely to pay for abortion costs, perhaps because no-test abortions can be less costly or because telehealth abortions are less likely to be covered by insurance.^25,26^ Other differences, including no-test patients being more likely to reside in rural areas and receive abortion medications by mail, highlight potential benefits of omitting in-person screening tests. Echoing other studies, patients in the no-test group were more likely to be White than those in the screening test group.^14,27^ Less medicalized models of care may be less likely to reach or interest patients of color.

In the complete case analyses, patients in the no-test group were more likely than those who obtained screening tests to be treated with additional medications. This may be attributed to a growing reliance on medications, such as additional misoprostol, to treat potential incomplete abortion, especially for patients who obtained their abortion care remotely and may face greater barriers to returning to the clinic.

The proportion of patients who had ED visits did not differ between study groups and was comparable to prior studies.^3,6,9,11,12^ ED visits involving treatment were more common in the no-test group, although absolute risks in both groups were low and RDs were small. No-test patients may have been more likely to reside further from the providing clinic and, therefore, may have been more likely to visit an ED for concerns necessitating in-person evaluation instead of the clinic, which is consistent with previous research.^28^ During ED visits, clinicians may have also been more likely to provide treatment if the patient did not have an in-person screening test due to concerns about incomplete abortion or ectopic pregnancy, at least initially when this model of care first became available. As no-test medication abortion providers gain more experience and refine care protocols, more recent data may not demonstrate this difference.^9^ One study that compared these models in 2020 to 2021 documented that the higher risk of procedural intervention and unplanned in-person follow-up visits found initially among no-test patients resolved over time.^12^

### Clinical implications

Although telehealth provision of medication abortion has increased, many providers of in-person medication abortion still require in-person screening tests. Based on our findings and the weight of the evidence from other studies, we recommend that clinicians can safely offer all eligible patients the option of no-test medication abortion, even in facilities with capacity for ultrasonography. Clinicians can rely on similar criteria, which include patient history to establish gestation and evaluate risk of ectopic pregnancy, to screen patients for no-test medication abortion eligibility.^1^ An update to the mifepristone label that clarifies Food and Drug Administration’s position that in-person preabortion tests are not necessary for all patients could accelerate the transition to this efficient, patient-centered model of abortion care.

### Research implications

Future research can investigate patient perspectives on the benefits and draw-backs of no-test models so that services can be tailored to underserved patients, including those in places where abortion is legally restricted.

### Strengths and limitations

This study is the largest to date to compare medication abortion provided with and without screening ultrasonography or pelvic examination, and the study groups were treated concurrently at the same clinics. Our results were consistent across several specifications of the analysis, all of which use robust methods to account for potential selection and confounding bias, supporting the credibility of our conclusions.

A major limitation is the high degree of loss to follow-up, particularly in the no-test group (42%). We used multiple imputations to estimate outcomes of lost to follow-up patients based on pretreatment characteristics, but this approach may be imperfect if post-treatment factors, such as treatment failure or adverse events, could influence follow-up. However, effectiveness in the no-test group appeared higher both in our complete case analysis and in the analysis that omitted data from the site with the highest loss to follow-up (which resulted in 76% follow-up), suggesting that our primary results may be conservative. Loss to follow-up after medication abortion is likely increasing due to the expansion of less medicalized abortion models,^2^ shifts in clinical guidelines away from universal follow-up,^15^ and the risks associated with recontacting clinics for patients residing in states with abortion bans. While follow-up is not clinically necessary, high loss to follow-up can exacerbate risks of selection bias and undermine research credibility. Researchers should investigate how to minimize and address loss to follow-up, including improving ascertainment of complications and analytic techniques to address missing data.

Second, complete abortion was determined by history alone in one-third of patients with that outcome. History-based outcome assessment is used clinically but has not yet been rigorously validated.^29,30^ If some patients determined by history to have complete abortions actually had ongoing pregnancies or subsequent interventions to complete the abortion, our analysis may overestimate effectiveness.

Third, because this study was a retrospective analysis of clinical data, some outcomes may have been under-estimated since they were not assessed systematically. Fourth, we lacked patient-level data reflecting the reasons why patients received each screening approach. While we used inverse-probability-of-treatment weighting to address confounding, the electronic medical records contained limited patient and abortion characteristics, potentially resulting in residual confounding.

## Conclusion

Since the 2022 *Dobbs* Supreme Court decision enabled 12 states to ban abortion entirely, remote abortion care models have become essential to meeting abortion demand where it remains legal and to reducing wait times in surge states. As of June 2024, 1 in 5 US abortions are provided by telehealth,^2^ demonstrating that no-test medication abortion can be delivered on a wide scale. Our study indicates that the mandatory ultrasonography requirements in 7 states where abortion remains legal^4^ are not evidence-based. Screening ultrasonography and pelvic examination can incur unnecessary costs, delay care, and be invasive. Expanding no-test medication abortion can increase access to abortion care without compromising effectiveness or safety.

## Supplementary Material

1

## Figures and Tables

**TABLE 1:** Characteristics of study sample with and without screening tests (*N*=2376)

Characteristics	Screening test group (*n*=649)	No-test group (*n*=1727)	*P* value^a^
Age at mifepristone provision, mean±SD	27.5±6.4	28.0±6.2	.070
12–18 years	17 (2.6)	30 (1.7)	.250
18–24 years	226 (34.8)	540 (31.3)	
25–29 years	172 (26.5)	485 (28.1)	
30–34 years	128 (19.7)	380 (22.0)	
35–47 years	106 (16.3)	292 (16.9)	
Pregnancy duration by last menstrual period, mean±SD	50.2±11.3	48.3±10.0	<.001
11–42 days	172 (26.5)	531 (30.7)	<.001
43–56 days	289 (44.5)	828 (47.9)	
57–63 days	95 (14.6)	222 (12.9)	
64–70 days	61 (9.4)	114 (6.6)	
70–77 days	32 (4.9)	32 (1.9)	
Race or ethnicity			
American Indian, Native American, or Alaska Native	12 (1.8)	18 (1.0)	<.001
Asian, Native Hawaiian, Pacific Islander	63 (9.7)	141 (8.2)	
Black	208 (32.0)	518 (30.0)	
Latinx	39 (6.0)	179 (10.4)	
White	205 (31.6)	734 (42.5)	
Multiple or another race or ethnicity	70 (10.8)	43 (2.5)	
Unknown	52 (8.0)	94 (5.4)	
Residence			
Urban	540 (83.2)	1339 (77.5)	.004
Suburban	39 (6.0)	151 (8.7)	
Rural	66 (10.2)	233 (13.5)	
Unknown	4 (0.6)	4 (0.2)	
Previous medication abortion			
No previous medication abortion	456 (70.3)	1283 (74.3)	.054
Any previous medication abortion	117 (18.0)	294 (17.0)	
Unknown	76 (11.7)	150 (8.7)	
Method of mifepristone dispensing			
In person	643 (99.1)	1131 (65.5)	<.001
Mailed	6 (0.9)	596 (34.5)	
Out-of-pocket contribution for abortion cost			
No out-of-pocket contribution	293 (45.1)	659 (38.2)	.002
Any out-of-pocket contribution	356 (54.9)	1068 (61.8)	
Clinic			
Clinic A	50 (7.7)	135 (7.8)	.974
Clinic B	299 (46.1)	786 (45.5)	
Clinic C	300 (46.2)	806 (46.7)	
Abortion outcome known			
Abortion outcome unknown	183 (28.2)	723 (41.9)	<.001
Abortion outcome known	466 (71.8)	1004 (58.1)	

*SD*, standard deviation.

a *P* value derived from Fisher’s exact tests.

**TABLE 2:** Outcomes after medication abortion with and without screening tests estimated from imputed, inverse-probability-of-treatment-weighted logistic regression

	Estimated proportion (95% CI)				
Characteristics	Overall (*n*=2376)	Screening test group (*n*=649)	No-test group (*n*=1727)	Risk difference (95% CI)	*P* value
Effectiveness					
Complete abortion without intervention^a^	93.4 (92.0, 94.9)	95.3 (90.6, 100.0)	93.4 (91.7, 95.0)	−1.9 (−6.9, 3.1)	.447

Intervention or continuing pregnancy^b^	6.6 (5.1, 8.0)	4.4 (0.1, 8.6)	6.7 (5.1, 8.3)	2.3 (2.3, 6.9)	.326

Aspiration or other procedure	4.0 (2.9, 5.1)	2.3 (0.0, 5.0)	4.3 (3.0, 5.6)	2.0 (1.0, 5.0)	.193

Prescribed >1600 mcg misoprostol, mifepristone, or other medications	2.5 (1.5, 3.5)	1.3 (0.0, 4.7)	2.7 (1.5, 3.8)	1.3 (2.3, 5.0)	.478

Suspected or confirmed ectopic pregnancy	0.48 (0.00, 1.14)	0.37 (0.00, 1.94)	0.50 (0.00, 1.29)	0.13 (1.56, 1.83)	.879

Confirmed continuing pregnancy	2.4 (1.5, 3.4)	2.2 (0.0, 5.7)	2.1 (1.1, 3.1)	−0.08 (−3.74, 3.58)	.967

Safety					

No major abortion-related adverse event	99.0 (98.2, 99.7)	99.8 (98.2, 100.0)	98.7 (97.8, 99.6)	−1.1 (−2.9, 0.7)	.234

Major abortion-related adverse event^b^	1.0 (0.3, 1.7)	0.19 (0.00, 1.59)	1.3 (0.4, 2.2)	1.1 (0.6, 2.8)	.198

Blood transfusion	0.89 (0.19, 1.59)	0.18 (0.00, 1.58)	1.1 (0.3, 2.0)	0.93 (0.73, 2.59)	.269

Major surgery, including surgical treatment of ectopic pregnancy	0.55 (0.00, 1.28)	0.15 (0.00, 0.73)	0.61 (0.00, 1.44)	0.46 (0.49, 1.42)	.339

Hospital admission	0.78 (0.11, 1.46)	0.17 (0.00, 1.25)	0.96 (0.14, 1.79)	0.80 (0.58, 2.17)	.255

Other outcomes					

Pregnancies found at follow-up to have been treated at >77 days gestation	0.43 (0.00, 1.08)	0.18 (0.00, 0.81)	0.50 (0.00, 1.30)	0.32 (0.66, 1.30)	.519

Emergency department visit	4.0 (2.8, 5.1)	3.9 (0.0, 8.3)	4.5 (3.1, 5.9)	0.56 (4.01, 5.13)	.810

Emergency department visit with treatment	1.7 (0.8, 2.6)	0.39 (0.00, 1.61)	2.1 (1.0, 3.2)	1.7 (0.1, 3.3)	.038

Inverse-probability-of-treatment weights include patient age, duration of pregnancy, race or ethnicity, urbanicity, prior medication abortion, method of mifepristone dispensing, and providing clinic.

Overall estimates draw from bivariate logistic regression models, while group-specific estimates and risk differences draw from inverse-probability-of-treatment-weighted logistic regression models.

*CI*, confidence interval.

a We estimated that 34% of complete abortions in the screening test group and 27% of complete abortions in the no-test group were determined by history alone;

b Categories are not mutually exclusive.

## AJOG at a Glance

### Why was this study conducted?

This study compared the safety and effectiveness of medication abortion care provided with and without screening ultrasonography or pelvic examination.

### Key findings

Effectiveness and safety were similar between patients who obtained screening ultrasonography or pelvic examination and those who did not. Emergency department visits involving treatment were more common in the no-test group.

### What does this add to what is known?

Clinicians can replace routine screening ultrasonography and pelvic examination with history-based screening without compromising the effectiveness or safety of their medication abortion care.
