# Supplementary material for: Effectiveness and safety of medication abortion with vs without screening ultrasonography or pelvic examination
Source: Am J Obstet Gynecol. Author manuscript; Available in PMC 2026 Jul 3. (PMC13328012; doi:10.1016/j.ajog.2025.06.013)
Supplement: 1 [file NIHMS2183378-supplement-1.pdf]

SUPPLEMENTAL FIGURE 1

Sample proportion with known abortion outcomes, by study group and sample characteristics

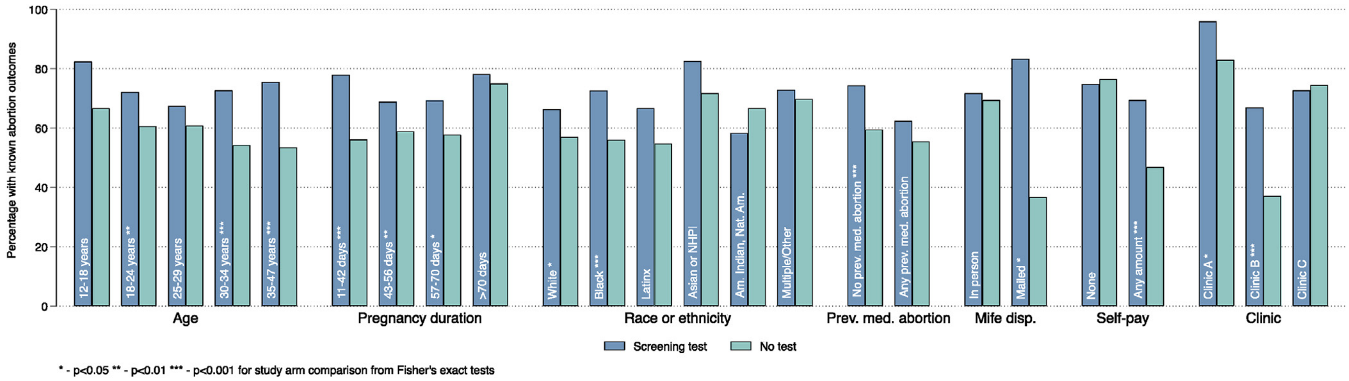

Abortion outcomes were more likely to be known in the screening test group within most strata of patient and abortion characteristics.

SUPPLEMENTAL TABLE 1

Complete case estimates of outcomes after medication abortion with and without screening tests estimated from unimputed, inverse probability of treatment—weighted logistic regression

	Overall (n=1470)		Screening test group (n=466)		No-test group (n=1004)			
	N	Estimated proportion (95% CI)	N	Estimated proportion (95% CI)	N	Estimated proportion (95% CI)	Risk difference (95% CI)	*P* value
Effectiveness								
Complete abortion without intervention[a]	1388	94.4 (93.2, 95.6)	443	95.8 (93.7, 97.9)	945	94.1 (92.3, 95.9)	−1.7 (−4.4, 1.1)	0.235
Intervention or continuing pregnancy[b]	82	5.6 (4.4, 6.8)	23	4.2 (2.1, 6.3)	59	5.9 (4.1, 7.7)	1.7 (−1.1, 4.4)	0.235
Aspiration or other procedure	52	3.5 (2.6, 4.5)	12	2.5 (0.8, 4.2)	40	3.6 (2.4, 4.9)	1.1 (−1.0, 3.2)	0.293
Prescribed >1600 mcg misoprostol, mifepristone, or other medications	26	1.8 (1.1, 2.4)	6	0.59 (0.00, 1.40)	20	2.1 (0.9, 3.2)	1.5 (0.1, 2.9)	0.040
Suspected or confirmed ectopic pregnancy	2	0.14 (0.00, 0.32)	1	0.10 (0.00, 0.73)	1	0.09 (0.00, 0.35)	0.00 (−0.68, 0.68)	0.996
Confirmed continuing pregnancy	28	1.9 (1.2, 2.6)	11	1.9 (0.5, 3.4)	17	1.7 (0.7, 2.7)	−0.26 (−2.02, 1.51)	0.777
Safety								
No major abortion-related adverse event	1462	99.5 (99.1, 99.8)	466	100.0 (99.4, 100.0)	996	99.2 (98.5, 99.9)	−0.81 (−1.74, 0.11)	0.084
Major abortion-related adverse event[b]	8	0.54 (0.17, 0.92)	0	0.00 (0.00, 0.60)	8	0.81 (0.11, 1.52)	0.81 (−0.11, 1.74)	0.084
Blood transfusion	6	0.41 (0.08, 0.73)	0	0.00 (0.00, 0.60)	6	0.63 (0.00, 1.28)	0.63 (−0.26, 1.52)	0.165
Major surgery, including surgical treatment of ectopic pregnancy	2	0.14 (0.00, 0.32)	0	0.00 (0.00, 0.60)	2	0.17 (0.00, 0.47)	0.17 (−0.50, 0.84)	0.619
Hospital admission	5	0.34 (0.04, 0.64)	0	0.00 (0.00, 0.60)	5	0.60 (0.00, 1.23)	0.60 (−0.27, 1.47)	0.179
Other outcomes								
Pregnancies found at follow-up to have been treated at>77 days' gestation	1	0.07 (0.00, 0.20)	0	0.00 (0.00, 0.60)	1	0.09 (0.00, 0.34)	0.09 (−0.56, 0.74)	0.789
Emergency department visit[c]	51	3.5 (2.5, 4.4)	10	2.7 (0.2, 5.2)	41	4.2 (2.8, 5.6)	1.5 (−1.4, 4.4)	0.312
Emergency department visit with treatment[c]	19	1.3 (0.7, 1.9)	1	0.00 (0.00, 0.59)	18	1.7 (0.8, 2.6)	1.7 (0.6, 2.7)	0.002

Inverse probability of treatment weights include patient age, duration of pregnancy, race or ethnicity, urbanicity, prior medication abortion, method of mifepristone dispensing, and providing clinic.

Overall estimates draw from bivariate logistic regression models, while group-specific estimates and risk differences draw from inverse probability of treatment—weighted logistic regression models.

[a] 35% of complete abortions in the screening test group and 28% of complete abortions in the no-test group were determined by history alone; [b] Categories are not mutually exclusive; [c] Denominator is n=468 in the screening test group and n=1006 in the no-test group.

SUPPLEMENTAL TABLE 2

Outcomes after medication abortion with and without screening tests among patients who obtained mifepristone in person estimated from imputed, inverse probability of treatment—weighted logistic regression

	Estimated proportion (95% CI)				
	Overall (n=1774)	Screening test group (n=643)	No-test group (n=1131)	Risk difference (95% CI)	*P* value
Effectiveness					
Complete abortion without intervention[a]	95.6 (94.4, 96.7)	95.3 (93.2, 97.4)	95.5 (93.9, 97.1)	0.20 (−2.49, 2.88)	0.885
Intervention or continuing pregnancy[b]	4.4 (3.3, 5.6)	4.7 (2.6, 6.9)	4.5 (2.9, 6.1)	−0.23 (−2.88, 2.43)	0.868
Aspiration or other procedure	3.1 (2.1, 4.1)	2.6 (1.0, 4.3)	3.5 (2.1, 4.9)	0.83 (−1.37, 3.03)	0.458
Prescribed>1600 mcg misoprostol, mifepristone, or other medications	1.2 (0.6, 1.9)	1.2 (0.0, 2.3)	1.4 (0.3, 2.4)	0.18 (−1.35, 1.72)	0.813
Suspected or confirmed ectopic pregnancy	0.45 (−0.07, 0.97)	0.44 (0.00, 1.41)	0.45 (−0.29, 1.19)	0.01 (−1.17, 1.19)	0.987
Safety	1.8 (1.0, 2.5)	2.4 (0.8, 4.0)	1.3 (0.4, 2.3)	−1.1 (−2.9, 0.8)	0.263
No major abortion-related adverse event					
Major abortion-related adverse event[b]	99.3 (98.8, 99.9)	99.8 (99.2, 100.5)	99.0 (98.0, 99.9)	−0.86 (−2.01, 0.30)	0.145
Blood transfusion	0.64 (0.11, 1.17)	0.20 (0.00, 0.94)	0.99 (0.07, 1.92)	0.79 (−0.38, 1.97)	0.185
Major surgery, including surgical treatment of ectopic pregnancy	0.51 (0.00, 1.03)	0.20 (0.00, 0.93)	0.81 (−0.06, 1.68)	0.61 (−0.49, 1.72)	0.278
Hospital admission	0.38 (−0.14, 0.90)	0.28 (0.00, 1.19)	0.48 (−0.30, 1.26)	0.20 (−0.97, 1.37)	0.732
Other outcomes	0.50 (−0.01, 1.01)	0.23 (0.00, 1.06)	0.74 (−0.10, 1.57)	0.51 (−0.65, 1.66)	0.390
Pregnancies found at follow-up to have been treated at>77 days' gestation					
Emergency department visit	0.39 (−0.13, 0.90)	0.28 (0.00, 1.15)	0.47 (−0.27, 1.21)	0.19 (−0.92, 1.30)	0.735
Emergency department visit with treatment	2.9 (2.0, 3.9)	1.9 (0.5, 3.3)	3.7 (2.3, 5.2)	1.8 (−0.2, 3.8)	0.074

Inverse probability of treatment weights include patient age, duration of pregnancy, race or ethnicity, urbanicity, prior medication abortion, method of mifepristone dispensing, and providing clinic.

Overall estimates draw from bivariate logistic regression models, while group-specific estimates and risk differences draw from inverse probability of treatment—weighted logistic regression models.

[a] Categories are not mutually exclusive; [b] We estimated that 21% of complete abortions in the screening test group and 32% of complete abortions in the no-test group were determined by history alone.

SUPPLEMENTAL TABLE 3

Outcomes after medication abortion with and without screening tests among patients at 2 of the 3 included clinics with ≥74% follow-up estimated from imputed, inverse probability of treatment–weighted logistic regression

	Estimated proportion (95% CI)				
	Overall (n=1291)	Screening test group (n=350)			
	Estimated proportion (95% CI)		No-test group (n=941)	Risk difference (95% CI)	*P* value
Effectiveness					
Complete abortion without intervention[a]	96.2 (94.9, 97.4)	95.5 (92.8, 98.1)	96.3 (94.8, 97.7)	0.83 (−2.20, 3.87)	0.591
Intervention or continuing pregnancy[b]	3.8 (2.5, 5.0)	4.3 (1.7, 6.9)	3.7 (2.2, 5.1)	−0.67 (−3.64, 2.29)	0.657
Aspiration or other procedure	3.0 (1.8, 4.1)	2.6 (0.5, 4.7)	3.2 (1.8, 4.6)	0.63 (−1.91, 3.17)	0.627
Prescribed>1600 mcg misoprostol, mifepristone, or other medications	0.81 (0.16, 1.46)	0.81 (0.00, 2.13)	0.83 (0.04, 1.63)	0.02 (−1.50, 1.55)	0.975
Suspected or confirmed ectopic pregnancy	0.38 (0.00, 0.97)	0.28 (0.00, 1.49)	0.41 (0.00, 1.11)	0.14 (−1.24, 1.52)	0.846
Confirmed continuing pregnancy	1.4 (0.6, 2.2)	1.8 (0.0, 3.6)	1.2 (0.3, 2.2)	−0.54 (−2.62, 1.54)	0.609
Safety					
No major abortion-related adverse event	99.3 (98.6, 99.9)	99.7 (98.6, 100.8)	99.1 (98.2, 99.9)	−0.61 (−1.98, 0.75)	0.379
Major abortion-related adverse event[b]	0.66 (0.03, 1.28)	0.21 (0.00, 1.22)	0.84 (0.03, 1.66)	0.63 (−0.64, 1.90)	0.332
Blood transfusion	0.50 (0.00, 1.07)	0.30 (0.00, 1.43)	0.60 (0.00, 1.33)	0.30 (−1.04, 1.64)	0.663
Major surgery, including surgical treatment of ectopic pregnancy	0.38 (0.00, 0.97)	0.28 (0.00, 1.49)	0.41 (0.00, 1.11)	0.14 (−1.24, 1.52)	0.846
Hospital admission	0.47 (0.00, 1.04)	0.26 (0.00, 1.38)	0.53 (0.00, 1.24)	0.27 (−1.02, 1.57)	0.682
Other outcomes					
Pregnancies found at follow-up to have been treated at >77 days' gestation	0.39 (0.00, 0.98)	0.28 (0.00, 1.45)	0.42 (0.00, 1.12)	0.15 (−1.19, 1.48)	0.830
Emergency department visit	2.9 (1.8, 4.0)	2.1 (0.2, 3.9)	3.4 (2.0, 4.8)	1.3 (−1.0, 3.6)	0.262
Emergency department visit involving treatment	1.4 (0.6, 2.1)	0.75 (0.00, 2.14)	1.7 (0.7, 2.7)	0.93 (−0.80, 2.66)	0.291

Inverse probability of treatment weights include patient age, duration of pregnancy, race or ethnicity, urbanicity, prior medication abortion, method of mifepristone dispensing, and providing clinic.

Overall estimates draw from bivariate logistic regression models, while group-specific estimates and risk differences draw from inverse probability of treatment–weighted logistic regression models.

[a] Categories are not mutually exclusive; [b] We estimated that 33% of complete abortions in the screening test group and 29% of complete abortions in the no-test group were determined by history alone.

SUPPLEMENTAL TABLE 4
Outcomes after medication abortion with and without screening tests

	Overall (n=1248)	Screening test group (n=374)	No-test group (n=874)	Risk difference (95% CI)	*P* value
	Estimated proportion (95% CI)				
Effectiveness					
Complete abortion without intervention[a]	93.9 (92.3, 95.4)	96.1 (94.1, 98.0)	93.8 (92.0, 95.6)	−1.3 (−4.1, 1.4)	0.341
Intervention or continuing pregnancy[b]	6.1 (4.6, 7.7)	3.9 (2.0, 5.9)	6.2 (4.4, 8.0)	1.3 (−1.4, 4.1)	0.341
Aspiration or other procedure	4.0 (2.7, 5.3)	2.5 (0.9, 4.1)	3.9 (2.6, 5.3)	0.92 (−1.18, 3.02)	0.391
Prescribed >1600 mcg misoprostol, mifepristone, or other medications	2.0 (1.1, 2.9)	0.73 (0.00, 1.52)	2.3 (1.1, 3.5)	1.2 (−0.2, 2.6)	0.088
Suspected or confirmed ectopic pregnancy	0.29 (0.00, 0.61)	0.39 (0.00, 1.00)	0.18 (0.00, 0.42)	−0.23 (−0.97, 0.51)	0.538
Confirmed continuing pregnancy	2.2 (1.3, 3.2)	1.8 (0.6, 3.1)	1.9 (0.9, 2.9)	−0.36 (−2.10, 1.38)	0.686
Safety					
No major abortion-related adverse event	99.4 (98.9, 99.8)	99.7 (99.1, 100.0)	99.1 (98.4, 99.8)	−0.55 (−1.52, 0.41)	0.259
Major abortion-related adverse event[b]	0.64 (0.17, 1.11)	0.30 (0.00, 0.88)	0.90 (0.18, 1.62)	0.55 (−0.41, 1.52)	0.259
Blood transfusion	0.53 (0.08, 0.98)	0.30 (0.00, 0.88)	0.72 (0.05, 1.40)	0.35 (−0.57, 1.27)	0.458
Major surgery, including surgical treatment of ectopic pregnancy	0.33 (0.00, 0.69)	0.30 (0.00, 0.88)	0.28 (0.00, 0.60)	−0.04 (−0.77, 0.68)	0.912
Hospital admission	0.59 (0.13, 1.06)	0.30 (0.00, 0.88)	0.71 (0.10, 1.32)	0.40 (−0.51, 1.32)	0.390
Other outcomes					
Pregnancies found at follow-up to have been treated at >77 days' gestation	0.24 (0.00, 0.54)	0.30 (0.00, 0.88)	0.17 (0.00, 0.40)	−0.13 (−0.83, 0.57)	0.719
Emergency department visit	3.7 (2.5, 5.0)	3.2 (0.3, 6.0)	4.7 (3.1, 6.4)	0.79 (−2.54, 4.12)	0.644
Emergency department visit with treatment	1.3 (0.6, 1.9)	0.30 (0.00, 0.87)	1.8 (0.8, 2.7)	1.3 (0.2, 2.4)	0.022

Estimates are drawn from inverse probability of treatment—weighted logistic regression using inverse probability of censoring weights to account for missing data.

Inverse probability of treatment weights include patient age, duration of pregnancy, race or ethnicity, urbanicity, prior medication abortion, method of mifepristone provision, and providing clinic.

Models included robust standard errors.

Overall estimates draw from bivariate logistic regression models, while group-specific estimates and risk differences draw from inverse probability of treatment—weighted logistic regression models.

[a] 35% of complete abortions in the screening test group and 28% of complete abortions in the no-test group were determined by history alone; [b] Categories are not mutually exclusive.

SUPPLEMENTAL TABLE 5
Outcomes after medication abortion with and without screening tests

	Overall (n=2376)	Screening test group (n=649)	No-test group (n=1727)	Risk difference (95% CI)	*P* value
	Estimated proportion (95% CI)				
Effectiveness					
Complete abortion without intervention[a]	93.4 (91.8, 95.1)	94.6 (88.2, 100.0)	93.6 (91.7, 95.4)	−1.0 (−7.7, 5.6)	0.756
Intervention or continuing pregnancy[b]	6.6 (4.9, 8.2)	5.4 (0.0, 11.6)	6.5 (4.6, 8.4)	1.1 (−5.4, 7.6)	0.739
Aspiration or other procedure	4.2 (2.8, 5.5)	3.2 (0.0, 7.9)	4.2 (2.7, 5.8)	1.00 (−3.93, 5.92)	0.691
Prescribed >1600 mcg misoprostol, mifepristone, or other medications	2.6 (1.4, 3.7)	1.3 (0.0, 4.8)	2.7 (1.4, 4.1)	1.5 (−2.3, 5.2)	0.441
Suspected or confirmed ectopic pregnancy	0.74 (0.00, 1.66)	0.76 (0.00, 3.18)	0.70 (0.00, 1.74)	−0.06 (−2.53, 2.40)	0.960
Confirmed continuing pregnancy	2.6 (1.5, 3.7)	3.0 (0.0, 8.2)	2.2 (1.0, 3.4)	−0.76 (−6.06, 4.54)	0.779
Safety					
No major abortion-related adverse event	98.9 (98.0, 99.8)	99.6 (97.3, 101.8)	98.6 (97.5, 99.7)	−0.92 (−3.45, 1.60)	0.473
Major abortion-related adverse event[a]	1.1 (0.3, 2.0)	0.43 (0.00, 2.29)	1.4 (0.3, 2.5)	0.96 (−1.15, 3.08)	0.370
Blood transfusion	1.0 (0.1, 1.9)	0.45 (0.00, 2.62)	1.2 (0.1, 2.3)	0.76 (−1.59, 3.10)	0.527
Major surgery, including surgical treatment of ectopic pregnancy	0.73 (0.00, 1.56)	0.50 (0.00, 2.88)	0.77 (0.00, 1.73)	0.28 (−2.25, 2.81)	0.830
Hospital admission	1.1 (0.2, 1.9)	0.51 (0.00, 2.62)	1.3 (0.2, 2.3)	0.76 (−1.53, 3.04)	0.516
Other outcomes					
Pregnancies found at follow-up to have been treated at >77 days' gestation	0.63 (0.00, 1.51)	0.48 (0.00, 2.65)	0.69 (0.00, 1.73)	0.20 (−2.10, 2.51)	0.863
Emergency department visit	4.1 (2.8, 5.4)	4.1 (0.0, 9.4)	4.7 (3.1, 6.2)	0.53 (−4.90, 5.95)	0.849
Emergency department visit with treatment	1.8 (0.8, 2.8)	0.33 (0.00, 2.16)	2.2 (1.0, 3.4)	1.9 (−0.3, 4.1)	0.090

Estimates are drawn from imputed logistic regression using monotone data imputation to account for missing data.

Inverse probability of treatment weights include patient age, duration of pregnancy, race or ethnicity, urbanicity, prior medication abortion, method of mifepristone provision, and providing clinic.

Models included robust standard errors.

Overall estimates draw from bivariate logistic regression models, while group-specific estimates and risk differences draw from inverse probability of treatment—weighted logistic regression models.

[a] We estimated that 30% of complete abortions in the screening test group and 27% of complete abortions in the no-test group were determined by history alone; [b] Categories are not mutually exclusive.
